# A prospective case series evaluating use of an in-line air detection and purging system to reduce air burden during major surgery

**DOI:** 10.1186/s13741-018-0104-9

**Published:** 2018-11-08

**Authors:** Yussr M. Ibrahim, Nicole R. Marques, Carlos R. Garcia, Michael Salter, Christopher McQuitty, Michael Kinsky, Mindy Juan, Achiau Ludomirsky

**Affiliations:** 1 Department of Anesthesiology, The University of Texas Medical Branch, Galveston, TX USA; 2 Department of Anesthesiology, Kadlec Regional Medical Center, Pasco, WA USA; 3 Department of Pediatrics, NYU Langone Health, School of Medicine , New York, NY USA

**Keywords:** Air embolism, Intravenous, ClearLine IV, Anesthesia, Fluid warmers

## Abstract

**Background:**

Intravascular air embolism (AE) is a preventable but potentially catastrophic complication caused by intravenous tubing, trauma, and diagnostic and surgical procedures. The potentially fatal risks of arterial AE are well-known, and emerging evidence demonstrates impact of venous AEs on inflammatory response and coagulation factors. A novel FDA-approved in-line air detection and purging system was used to detect and remove air caused by administering a rapid fluid bolus during surgery.

**Methods:**

A prospective, randomized, case series was conducted. Subjects were observed using standard monitors, including transesophageal echocardiography (TEE) in the operating room. After general anesthesia was induced, an introducer and pulmonary artery catheter was inserted in the right internal jugular to administer fluids and monitor cardiac pressures. Six patients undergoing cardiac surgery were studied. Each patient received four randomized fluid boluses: two with the in-line air purging device, two without. For each bolus, a bulb infuser was squeezed three times (10–15 mL) over 5 s. The TEE was positioned in the mid-esophageal right atrium (RA) to quantify peak air clearance, and images were video recorded throughout each bolus. Air was quantified using optical densitometry (OD) from images demonstrating maximal air in the RA.

**Results:**

All subjects demonstrated significantly lower air burden when the air reduction device was used (*p* = 0.004), and the average time to clear 90% of air was also lower, 3.7 ± 1.2 s vs. 5.3 ± 1.3 s (*p* < 0.001).

**Conclusion:**

An air purging system reduced air burden from bolus administration and could consequently reduce the risk of harmful or fatal AEs during surgery.

## Background

Intravascular air embolism (AE) occurs when undesired air enters the venous or arterial circulation, typically during medical procedures (Mirski et al. 2007; McCarthy et al. 2017). Once air enters the patient, symptoms range from subtle physiologic changes to potentially catastrophic events. The impact of AEs depends upon factors including the patient’s physiology, size of the air mass, and the path air takes through the anatomy (Mirski et al. 2007; Orliaguet and Martin 2000; Brull and Prielipp 2017). AEs may enter the vasculature during major surgeries such as neurosurgery, or during less complex procedures including administration of medications, fluids, or blood products through intravenous tubing, intravenous catheter placement, or during diagnostic procedures (Bayliss et al. 2014). AEs may be comprised of atmospheric air or medical gases including nitrous oxide, carbon dioxide, helium, and nitrogen (Mitchell et al. 2000; McGrath et al. 1989).

When entering the venous system in healthy individuals, air is usually broken up in the capillary bed of the lungs. However, studies demonstrate that entrapment of venous air in the pulmonary microcirculation can decrease gas exchange and cause pulmonary vascular obstruction, potentially leading to release of vasoactive mediators. Ultimately, this may result in cellular injury and lung edema (van Hulst et al. 2003). Larger volumes of venous air can increase pulmonary artery pressure and right ventricular strain, resulting in systemic cardiovascular collapse (Muth and Shank 2000; Agarwal et al. 2009).

A paradoxical AE occurs when venous air enters arterial circulation, such as in the case of an atrial or ventricular septal defect (ASD and VSD). In cases of large venous AE, the filter capacity of the pulmonary capillary bed becomes overwhelmed and air may translocate to the arterial circulation, more often in patients with a patent foramen ovale (PFO) as well as an ASD or VSD, thereby allowing direct air passage from the venous to arterial system (Girard et al. 2003; Park et al. 2010). This phenomenon provides considerable risk since most PFOs are undiagnosed and occur in 10 to 25% of the population (Mirski et al. 2007; Brull and Prielipp 2017; Vesely 2001; Foster et al. 2003). The closer the air entry to the right heart, the lesser the volume of air required to cause fatality (McCarthy et al. 2017). Systemic inflammatory response syndrome (SIRS) can also result from AEs (Kapoor and Gutierrez 2003; Hsieh et al. 2009).

Clinicians are trained to reduce AE risk by priming intravenous tubing and use devices including Luer-lock connectors, filters, and modern infusion systems which may alarm on detection of AE. Despite this, AEs have been reported in cases of emergency, in cases of home intravenous treatment, or due to failures of pump air-in-line sensors or other device malfunctions (Laskey et al. 2002; Wilkins and Unverdorben 2012; Paul Pelletier and Fisher 2017).

The use of pressure infusion devices and fluid-warmers increases AE risk. Pressurized infusion devices rapidly deliver intravenous (IV) fluid, and air entrainment can occur from errors in manual setup. Pressurized infusion devices have limited and variable capability to detect and remove air during infusions (Schnoor et al. 2004; Zoremba et al. 2011; Woon and Talke 1999; Smith et al. 2001). As fluid is warmed, micro- and macro-bubbles dissipate from the fluid as it is heated. Air from IV tubing can be due to direct air injection or delivered into the circulation when IV fluid is heated. Specifically, this occurs when fluids are actively heated from room temperature (as often case with crystalloid fluids) or from chilled (blood and blood products). The concentration of dissolved air is based on pressure and temperature. According to Henry’s law, the dissolved gas is a function of temperature, where, as temperature increases, dissolved gas/air comes out of solution in form of bubbles. Outgassing and air production when heated to 41 °C can yield 1–5 mL per liter of fluid (with colder fluids such as blood products yielding higher volumes) (Varga et al. 2016). While in-line filters help remove particulate contamination and entrapped air, they may increase flow resistance, limiting usefulness when administering rapid fluid boluses. AEs are costly events. Data from the American Society of Anesthesiologists Closed Claims Project showed 100% of claims for venous AE resulted in payment with a median of $325,000 (Brull and Prielipp 2017).

A novel device ClearLine IV™ (ClearLine MD, Woburn, MA, USA) is cleared by the FDA for automatic detection and removal of in-line air at a minimum volume of 25 μL, a quantity below that is shown to cause patient harm. This pilot study was conducted to quantify air entering a patient during bolus infusion and to test our hypothesis that ClearLine IV successfully identifies and reduces patient’s air burden during major surgery.

## Methods

The study protocol was approved by the Institutional Review Board (IRB), and all patients provided written informed consent prior to undergoing study procedures. Eligible patients included adults, ages 18 to 80, undergoing cardiac surgery, who were not pregnant at the time of screening. Major exclusions include malignant hypertension, esophageal disease, severe heart failure, pulmonary hypertension, and those undergoing emergency surgery. Cardiac surgery patients were chosen since they normally undergo monitoring that would permit the quantification of air necessary for the study.

**Fig. 1:**
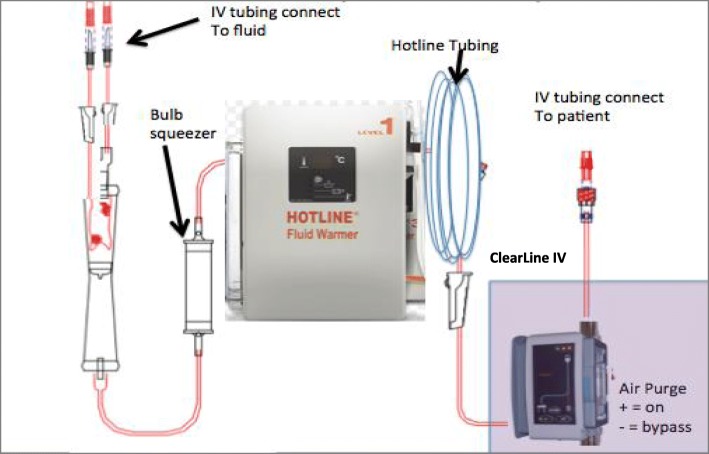
Setup for IV fluid administration. Fluid administration setup with ClearLine IV. Ultrasound sensing technology detects and diverts air from the IV line to a collection bag through opening of a valve to a waste reservoir. The fluid is redirected to the patient line after the air is entrapped through another valve opening or can be bypassed (blue lines). IV: intravenous

The study was a case series of prospective, randomized, crossover design conducted at a single center. Enrollment occurred between November 2016 and January of 2017. Following consent, standard of care monitors and central lines were used to administer general anesthesia. After induction of anesthesia, an 8.5-Fr Cordis introducer with a pulmonary artery catheter (Edwards Lifescience, Irvine, CA, USA) was inserted in the right internal jugular to administer fluids and monitor cardiac pressures. Warmed crystalloid was delivered using HOTLINE® (HL90 Hotline Level 1, Smiths Medical, Rockland, MD, USA) with a blood filter and pressure pump (Baxter, Deerfield, IL, USA) in-line with the ClearLine IV (treatment) or via tubing, bypassing the ClearLine IV system (control) (Fig. 1).

ClearLine IV consists of a control unit and a sterile disposable component (infusion catheter). The device connects to infusion lines and uses ultrasonic sensors to detect air volumes as small as 25 μL. Once detected, the device diverts fluid flow to a waste collection bag and a second ultrasonic sensor detects when the air has been removed, at which time the controller automatically restarts the infusion flow to the patient. This device only briefly (seconds) interrupts the intravenous infusion and results in less than 10 mL fluid lost per air-removal cycle.

For each bolus delivery, a pressure infuser (bulb) was squeezed three times, delivering a volume of 10 to 20 mL each time over the course of 5 s per single bolus, as is done per standard of care during cardiac procedures. Flow rates were measured by a modified scale that measured the change in mass (grams) over time (min). Each IV fluid bag was suspended from the scale and zeroed prior to bolus. We assumed that each 1 g was equivalent to 1 mL. Each bolus was followed by a 60- to 90-s pause. Each patient received four randomized boluses: two with ClearLine IV and two control boluses. Bolus order was assigned using a randomized block design to determine assignment of the first fluid bolus. Thereafter, the first and third and the second and fourth boluses were paired. Transesophageal echocardiography (TEE) (Vivid GE, Milwaukee WI) was used throughout the procedure.

When fluids are heated by warming systems, such as the Hotline, small amount of air is generated secondary to decrease in gas solubility with increasing temperature. Squeezing the bulb several times provides high infusion rates that also generate small bubbles. This is similar to rapidly injecting agitated saline to check for an inter-atrial shunt. Thus, by rapidly mixing the syringe, small air bubbles come out of solution and can be observed via TEE. The amount of air bubbles produced would be on a much larger scale by squeezing the bulb and warming the fluid.

### Quantification of air burden

While TEE is the most sensitive means to determine the presence of air, to our knowledge, there is no specific ultrasound software available to quantify the amount of air in the heart. We therefore used a commercially available software from NIH (ImageJ software for MAC, NIH, Bethesda, MD, USA) to determine the maximal amount of air present in the right atrium. As this study is first to describe this methodology to assess and quantify air in the heart using this technique, we believe that this approach is novel, since ultrasound naturally has a contrast between air and fluid, which allows for visual patterns to be at least semi-quantifiable. Video sections of the right atrium (RA) and right ventricle (RV) were obtained by TEE positioned in a mid-esophageal RA view for peak air amount and clearance. TEE was chosen both because it is routinely available in the hospital and it is the most sensitive and specific indicator of intravascular air. TEE images were video recorded throughout each bolus of warmed crystalloid.

**Fig. 2:**
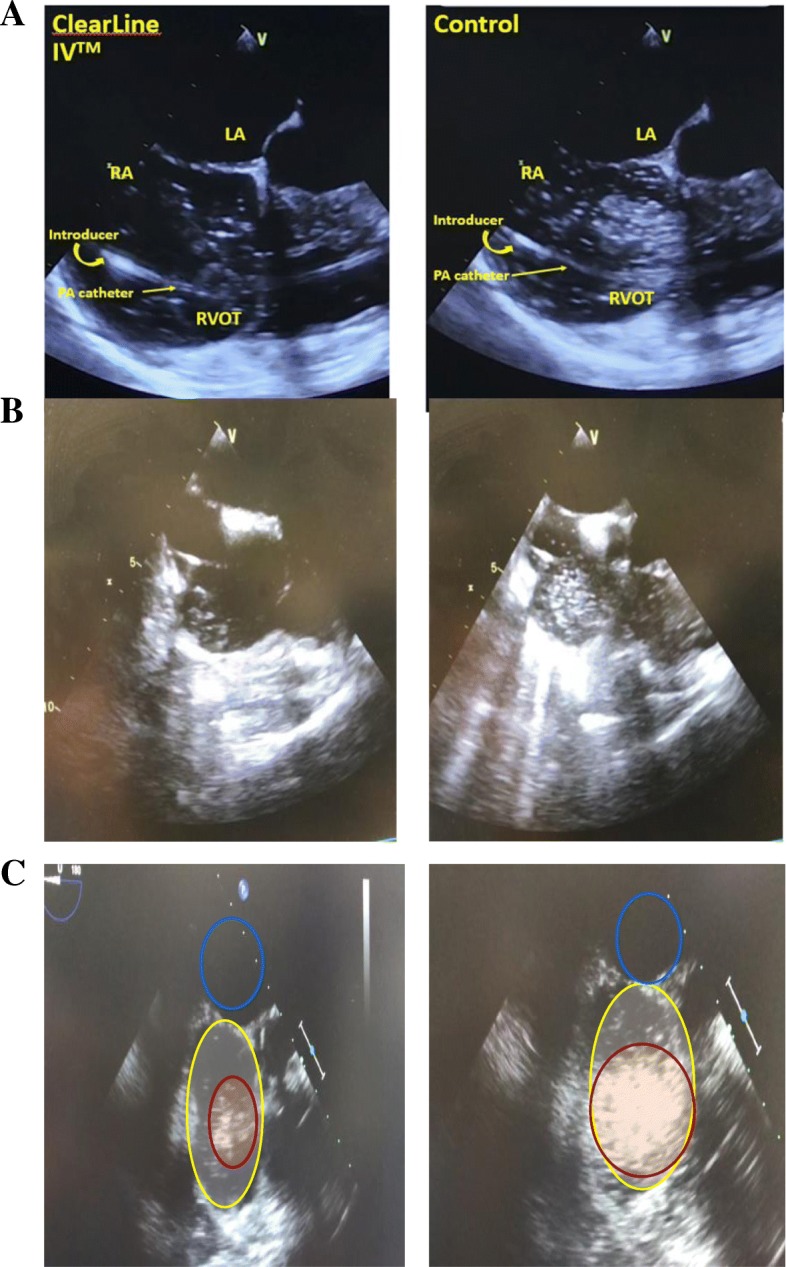
Air quantification in the right atrium by optical densitometry (OD). ClearLine IV (left) versus control (right). **a**–**c** TEE images during bolus. LA: left atria, RA: right atria, RVOT: right ventricular outflow tract

Images throughout bolus administration were reviewed, and the image at the time of maximal air in the chambers was selected and screen locked for each bolus. A control image for each patient was established where no air was present. The left atrium was used as negative control as it had similar contrast values but no air present. The images were then uploaded, the RA was outlined, and the amount of air was quantified using optical densitometry (OD) software (ImageJ software for MAC, NIH, Bethesda, MD, USA). Mean OD was determined by subtracting the peak image from the control image (Fig. 2). Mean OD values for the control versus the ClearLine IV group were determined by two independent investigators and then averaged for each subject. Air clearance, defined as the time that 90% of air from the RA was eliminated, was also calculated.

### Statistical analysis

#### Sample size estimate

Previous animal studies showed that the ClearLine IV could reduce air entrainment by at least 50%. Thus, we calculated that air in the right atrium without ClearLine IV to maximally cover 50% of the right atrium, ± 20% standard deviation. Our preliminary animal studies (data not shown) demonstrated that this could be reduced by half. Thus, we tested the hypothesis that ClearLine IV could reduce air entrainment to 25% with a 20% standard deviation. Based on a power of 0.80 and alpha of 0.05, *n* = 6 subjects per group would be needed to test this statistical hypothesis. Data were collected and entered in Excel for statistical analysis. Means and standard deviations for the air burden and air clearance were calculated by two independent investigators, and the final analysis included the values of both investigators. A paired-sample *t* test was conducted to compare the means of the ClearLine IV and control groups with the significance level set at *p* = 0.05.

## Results

**Fig. 3:**
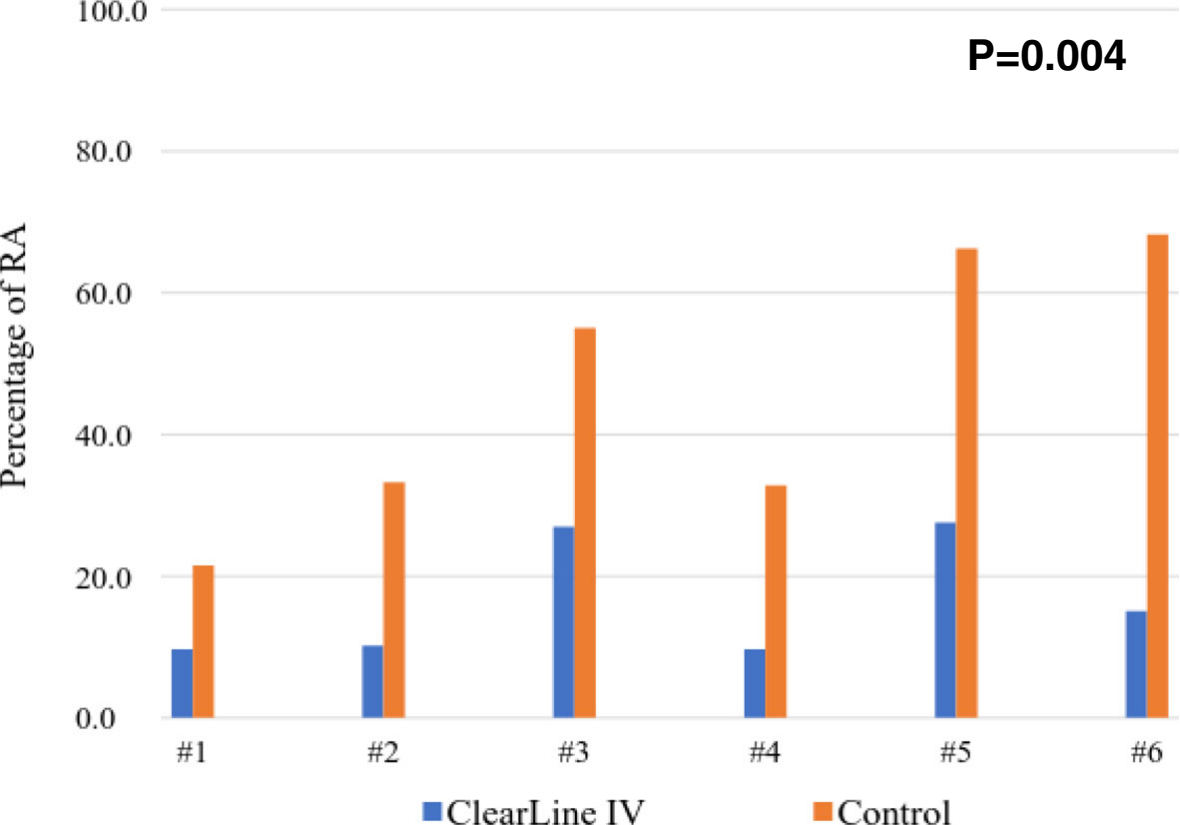
Air quantification as measured in the right atrium (RA) by TEE images and optical densitometry (OD). Less air was quantified in the RA following boluses administered in-line with the ClearLine IV system. TEE: transesophageal echocardiography

Six subjects completed the study, five males and one female (Table 1). Air quantification was conducted by two independent investigators, and the results in all cases had less than 10% error between the investigators. The flow rates of fluid boluses were similar, 145 ± 22 mL/min for ClearLine IV and 137 ± 45 mL/min for control (Table 2). Subjects demonstrated significantly less mean RA air in the ClearLine IV group (OD = 16 ± 3%) compared with those in the control group (OD = 46 ± 7%), *p* = 0.004 (Fig. 3). Air clearance time was significantly lower with the use of ClearLine IV (3.7 ± 1.2 s) compared to control (5.3 ± 1.3 s), *p* < 0.001.

**Table 1:** Baseline demographics

	Gender	Age	Race/ethnicity
Subject 1	Male	72	White/non-Hispanic
Subject 2	Male	61	White/non-Hispanic
Subject 3	Male	60	Black/non-Hispanic
Subject 4	Male	65	White/non-Hispanic
Subject 5	Male	49	White/Hispanic
Subject 6	Female	73	White/non-Hispanic

**Table 2:** Air quantification by optical densitometry (OD)

	ClearLine IV mean ± SD	Control mean ± SD	*p* value
Fluid flow rates (mL/min)	145 ± 22	137 ± 45	NS
Mean RA air by OD (%)	16 ± 3	46 ± 7	*p* = 0.004
Air clearance time (s)	3.7 ± 1.2	5.3 ± 1.3	*p* < 0.001

## Discussion

Air embolism is an uncommon but potentially life-threatening event for which prompt diagnosis and treatment can lead to a better patient outcome. Air embolism formation requires a connection and pressure gradient between the blood vessel and the gas. The air bubbles have the deteriorating effect as they can migrate to different vital organs, thus negatively affecting patient morbidity and mortality. Most air emboli in the vascular system are iatrogenic in nature (Mirski et al. 2007). Venous air emboli may occur during pressured venous infusion and/or catheter manipulation such as during insertion of intravascular catheters, including peripheral and central venous cannula and diagnostic and therapeutic catheters. Air embolism in the venous circulation usually travels into the right atrium and ventricle and then reaches the pulmonary circulation. Since a large percentage of the population carries the diagnosis of patent foramen ovale (PFO), a risk of paradoxical embolus is present when the air bubble travels across the PFO into the systemic circulation, hence reaching vital organs like the brain and kidneys. Arterial air embolism most often occurs during significant invasive procedures. These include bronchoscopy, craniotomies, esophagogastroscopy, and lung and liver biopsies.

The literature reports an increased incidence of cases of air emboli during cardiac surgeries that require the patient to be on the heart-lung machine, extracorporeal membranous oxygenation (ECMO), and/or left-sided assist devices (LVAD). In the case of arterial air embolism, the air may flow in a retrograde way via different arteries and reach the cerebral circulation. If the air reaches the left ventricle, there is a possibility that the air will enter the coronary arteries and cause significant cardiac ischemia and arrhythmia.

During the last two decades, a breakthrough has been achieved in the treatment of ischemic and congenital heart disease via a transcatheter approach. These procedures are high-risk and invasive in nature. Currently, we can perform balloon dilation of small vessel and stenotic valves among other procedures. The technology also provides the interventionists with tools to perform atrial and ventricular septal defect closure as well as valve repair and replacement via a catheter approach. The duration of these complex and innovative procedures is long and requires multiple arterial and venous axes. Continuous flow is necessary to keep these lines, arterial and venous, patent and ready for catheter manipulations. Different maintenance solutions including blood products and both cardiac and anesthetic medication are administered to the patient via these lines. These manipulations significantly increase the risk of air embolism in this population of patients.

A large air embolism may cause catastrophic events and lead to death regardless if it is in the systemic or pulmonary circulations. In the venous system, large amounts of air (300–500 mL) may lead to RV failure, cardiogenic shock, and death. In contrast, small amounts of air in the arterial circulation can be fatal and lead to coma, seizures, myocardial ischemia and infarction, arrhythmia, and heart failure (Toung et al. 2001).

It is known that even small amounts of air emboli have negative effects on different tissues. A surge of inflammatory response occurs as a marker for physiological tissue impairment. There is also non-obvious damage caused by bubbles in the microcirculation, which include tissue ischemia, micro-thrombosis, endothelial damage, and cytokine release.

Varga and colleagues introduced the concept of “The Partially Invisible Phenomenon” which was noticed also by other investigators (Varga et al. 2016). This phenomenon describes the potential harm caused by outgassing from intravenous fluid or cold blood products during warming to normal body temperature. The dissolved gas is invisible and can be administered unintentionally and undetectably. Since many institutes are using warmers as a standard of care in their patients’ circuits, it is crucial that we pay serious attention to potential air embolism symptoms. It requires close monitoring of cardiac and brain activity (Barak and Katz 2005).

The objective of this study was to quantify air entering a patient during bolus infusion during surgery and to test whether ClearLine IV reduced patient’s air burden. Several factors related to fluid warming and infusion place patients undergoing major surgery at risk of AE. Fluid warming and pressurized infusion devices are common in the operating room setting, and studies have shown that these devices increase AE risk (Orliaguet and Martin 2000; Zoremba et al. 2011). Further, fluid flow rates create variability in air introduction, as the volume of air introduced increases as the infusion flow rate decreases. Other factors may unpredictably impact the air volume introduced, as gas bubbles are dynamic, and, as they flow and come into contact with other emboli, may combine to form larger emboli more likely to cause patient harm (Van Liew and Burkard 1995). Taken together, these findings suggest that any quantity of air should be removed, if possible, to mitigate risk. In-line filters, while widely accepted for air and particle removal, are unable to remove all air from circulation and increase resistance in the line, making their utility challenging, particularly in the setting of bolus infusion (Herbst and Najm 2012). While some fluid warming devices can detect air, the air elimination capability of these systems demonstrates variable results, particularly in the setting of pressurized infusions (Pelletier and Fisher 2017; Schnoor et al. 2004; Zoremba et al. 2011a; Woon and Talke 1999; Smith et al. 2001). ClearLine IV has been shown to successfully detect air using an ultrasound sensor, as small as 25 μl, and thereby eliminate air from entering into IV tubing. While ClearLine IV can eliminate large volumes of air (> 50 mL), flow rate is the limiting factor. Specifically, the ClearLine IV sensor triggers a diverter “switch” enabling air to be bypassed. ClearLine IV can accommodate and remove air semi-continuously as long as the flow rate does not exceed 400 mL/min (product safety studies). Commercialized IV fluid pumps on the market rarely deliver fluid exceeding this rate. Few infusion pumps exceed 100 mL/min. Prior to this clinical study, we tested the Clearline IV in our lab. Using a similar setup (bulb-squeezer pressure pump connected to Hotline) shown in “Methods”, a Doppler flow probe (in-line, Transonic flow meter, Ithaca, NY, USA) was placed on the IV tubing connected to an 8.5-Fr Cordis line. We demonstrated that the maximum flow rate during a rapid bolus using the Hotline with a bulb squeezer did not exceed 250 mL/min. We appreciate that devices for large-volume resuscitation, e.g., Level 1 (under high driving pressure) or Belmont rapid infuser, can deliver fluids at 500 mL/min. Both of these commercialized products have a separate air trap device. In these cases, using the Belmont or Level 1, the flow rate would exceed the capability of the ClearLine IV device.

Therefore, identification of additional technological options to mitigate the residual risk of AE is clinically important and motivated us to conduct this investigation of the ClearLine IV system.

The results of our investigation confirm that during major surgery, particularly in the setting of fluid warming devices and pressure and bolus infusions, quantifiable AEs are introduced into the patient’s vasculature. While left-sided air is common during cardiac surgery and enters the cerebral circulation and other systemic organs, the potential presence of a PFO, ASD, or VSD and the risk of right-sided AEs offer additional clinical risk. Further, increasing evidence suggests that AEs of any size can result in patient harm. In fact, neuropsychological injury arising from small volumes of air following cardiopulmonary bypass remains one of the most serious and costly complications of the procedure. These micro-emboli arise from surgical and manual manipulation of the heart and arteries and the components of the extracorporeal circuit (Lou et al. 2011).

While none of the subjects in this study experienced a detectable adverse experience from the air introduced, patients who are compromised or have a PFO/ASD/VSD could be at higher risk for negative impact of AE. Special consideration can be taken for patients with a known PFO to prevent paradoxical embolization, although given the high rate of undiagnosed PFO and high cost of AE, the ClearLine IV system may provide an important role in all surgical patients to avoid this potential risk.

### Limitations

The sample size for this study was small (*n* = 6). The study was not designed to impact the outcome but rather was performed to determine if the ClearLine IV system could significantly reduce the delivery of intravenous air into the right heart. While the series was randomized, the investigators were not blinded when they quantified the air in the RA images, since audiovisual files were created to distinguish the timing of sequential fluid boluses. The investigators of this study were not aware of any commercially available software to objectively quantify air in the RA. While the OD (optical densitometry) methodology is not officially validated for determining intra-atrial air, the use of the ImageJ software provided a well-known method for calculating OD. Further, the fact that two investigators scored all images independently and measures were within 10% of each other reduces the risk of error or bias introduced in this process. We also recognize that we are using two-dimensional measurements, and “air”, which is not lucid via ultrasound, will be reduced in the far field (away from the probe). Thus, air in the far field would not be as reflective. However, we suggest that this limitation would be present in both assessments with and without the ClearLine IV group. Although this study did not measure the effects of asymptomatic air emboli (e.g., inflammatory response to micro-air emboli without evidence of hemodynamic collapse), it is possible that this factor would be attenuated given the threefold reduction in overall air entrainment with the ClearLine IV system.

## Conclusion

Our study found that use of ClearLine IV in-line with the circuit significantly reduced the quantity of RA air when delivering a warmed solution at a fast rate without changing the rate of bolus infusion or creating resistance in the circuit. Subsequently, the clearance time for air was also significantly faster in the ClearLine IV circuit. This device could thereby reduce risk of harmful and potentially fatal AEs. While the total volume of air removed was relatively small in these cases (3–7 mL), given the nature of rapid bolus, error could result in administration of greater volumes of air during these procedures and even small amounts of air could result in harm. The results of this study demonstrate the utility of the ClearLine IV system to reduce the amount of air introduced into the patient when delivering a warm solution at a fast rate during major surgery. Further studies are warranted to assess efficacy in preventing harmful sequelae of air embolism such as coagulation changes, systemic inflammatory response syndrome (SIRS), pulmonary edema, and ischemia.

## Abbreviations

AE: Air embolism
ECMO: Extracorporeal membranous oxygenation
IRB: Institutional Review Board
LVAD: Left-sided assist devices
OD: Optical densitometry
PFO: Patent foramen ovale
RA: Right atrium
RV: Right ventricle
TEE: Transesophageal echocardiography
